# Measuring activities in tobacco control across the EU. The MAToC

**DOI:** 10.1186/1747-597X-1-9

**Published:** 2006-04-18

**Authors:** Jochen René Thyrian, Ulrich John

**Affiliations:** 1 Institute for Epidemiology and Social Medicine, Ernst-Moritz-Arndt University of Greifswald/Germany, Ernst-Moritz Arndt University of Greifswald, Walther-Rathenau-Str. 48, 17489 Greifswald, Germany

## Abstract

**Background:**

Objectives of this study are (a) to develop a comprehensive and economic tool to estimate tobacco control (TC) activities in single EU member states, (b) to compare TC activities between member states of the EU. This article provides the questionnaire and gives a benchmark of EU member states according to their perceived TC activities. Methods: An international workshop was specifically initiated to develop the questionnaire "Measuring Activities in Tobacco Control (MATOC)". TC experts from 8 European countries participated and chose 40 items to cover 11 general topics of TC. At the World Conference of Tobacco or Health in Helsinki 2003 participants were asked to fill out the questionnaire. N = 142 participants from EU-member states returned questionnaires.

**Results:**

Subjects from the tobacco field in Finland gave the highest TC values to their country, followed by Sweden, Ireland, the UK and the Netherlands. The least active countries in TC were Greece and Germany, behind Austria, Spain, Belgium and Portugal. Italy, France and Denmark constituted the middle field.

**Conclusion:**

The MATOC provides a profile of TC across European countries and delivers results that are plausible and fit into the existing findings. The data presented here fulfils the purpose to illustrate what is possible with the MATOC and underlines the value of such an approach in delivering information for policy makers and TC advocates how TC is perceived in each country. Yet, further validity testing is necessary, the number of experts per country differs and is partly rather small. Further research with the MATOC should encounter these limitations. The procedure though could serve as model of practice for alcohol and legal drug policy as well.

## Background

In Europe, current tobacco control (TC) research faces a series of problems (a) country TC profiles in different countries describe and compare only single TC measures [1,2]. (b) TC is a very comprehensive and rapidly changing field. Gathering comprehensive information about TC is time consuming, integration of data across countries is hard to accomplish, and updating the data is a constant need. While European Union (EU) wide legislation is adopted across countries in a similarly manner, country specific regulations differ in their enforcement and their implementation.

The goal of this study was to (a) develop a tool to estimate TC activities in single EU member states. Demands of this tool are to be comprehensive in its scope, economic in its data assessment and valid in describing a country's TC, (b) to compare the perceived TC activities of EU-member states according to this tool.

To accomplish these goals, we developed a brief questionnaire for expert ratings. The advantage of expert ratings is three-fold: 1. Expert ratings are an economic way to gather information about TC activities. 2. Expert ratings, we assume, include knowledge about what is "no activity at all" and what is "desirable activity". 3. Expert ratings may help to fill the gap between the knowledge what is desirable in TC and what is realised in a country. When, for example, an age restriction on smoking cigarettes exists, expert ratings may give a valuable estimate about the degree to which this law is realised. Previous research has indicated that questionnaires or expert ratings can be used to a satisfying degree to assess the quality of TC policies [3,4].

## Methods

The item pool for the MAToC (Measuring Activities in Tobacco Control) was generated during an international workshop specifically initiated to develop the questionnaire. TC experts from 8 European countries participated and the 11 topics chosen to be covered by 40 items of the questionnaire were: taxing, smuggle, product control, smoking cessation, media, protection from exposure to environmental tobacco smoke (ETS) which means second hand smoke, health care, research, politics, population, and prevention. These were the topics that were agreed on by the experts to play a vital role across EU countries and that have shown efficacy, according to the experts, in changing a country's smoking rate or smoking climate. Advertising was a topic too, but was excluded in this analysis due to item wording that was misunderstood by many respondents. The questionnaire includes questions about the respondent's country, his smoking status, field of work (education, treatment, research, policy) and his sex. Response patterns range from yes/no/don't know answers to 5-point Likert scaled items indicating agreement to the statement from "not at all" (1) to "absolutely" (5). The difference in response pattern reflects the difference in the required information. The 5-point Likert scale allows respondents to rate to what extent a statement is implemented in a country, while Yes/No questions were used in items where the MAToC asks for facts or activities reflected in existing legislation (like: Are health warnings required?). The tobacco-control related items of the MAToC, their response pattern and the categorisation into subscales are illustrated in Table 1.

**Table 1:** The MAToC: items, response pattern and subscale of tobacco control

Subscale	internal consistency^A^	Item	Response pattern
Taxing	.566	1. Taxing cigarettes is used by the government as a tobacco control measure	dichotomous
		2. At least some tobacco taxes are specifically used to help fund the health care system	dichotomous
Smuggling		3. There is an effective strategy for combating smuggling of cigarettes	dichotomous
Prevention	.698	4. Smoking of cigarettes is not allowed to children/adolescents under a certain age	Dichotomous
		4.a. These restrictions are effectively enforced	5-point Likert scale
		5. Sale of cigarettes is not allowed to children/adolescents under a certain age	Dichotomous
		5.a. These restrictions are effectively enforced	5-point Likert scale
		6. Education on the dangers of tobacco use are part of the school curriculum	5-point Likert scale
Product Control	.609	7. Health Warnings must be displayed on cigarette packs	dichotomous
		8. Tar and Nicotine yields of cigarettes must be displayed on the packets	dichotomous
		9. There are restrictions on the tar and nicotine yields of cigarettes	dichotomous
Smoking Cessation	.442	10. Smokers have ready access to effective help with smoking	5-point Likert scale
		11. Efforts are made to encourage people to use effective smoking cessation treatment	5-point Likert scale
		12. There are systems to try to ensure the quality of treatment services aimed at helping smokers to stop	5-point Likert scale
		13. It is expensive for smokers to obtain treatment to help them stop smoking	5-point Likert scale
Protection from ETS	.843	14. – 18.e. There are effective restrictions on smoking in: (5 separate items) Schools, Worksites, Public places, Hospitals, Bars	5-point Likert scale
Media Support	.698	19. Advertising campaigns regularly appear in the media of dangers of smoking	5-point Likert scale
		20. The media give adequate publicity to the health effects of smoking	5-point Likert scale
		21. The media support the anti-smoking agenda	5-point Likert scale
Health Care System	.887	22. Doctors support the anti-smoking agenda	5-point Likert scale
		23. Nurses support the anti-smoking agenda	5-point Likert scale
Research	.787	24. Tobacco control initiatives are well funded	5-point Likert scale
		25. Research aimed at reducing smoking is well funded	5-point Likert scale
		26. There are many tobacco researchers in my country	5-point Likert scale
Politics	.683	27. There is an explicit tobacco control strategy	5-point Likert scale
		28. The government is strongly anti-smoking	5-point Likert scale
Population	.629	29. Compliance with tobacco regulations is good	5-point Likert scale
		30. There is a strong anti smoking ethos	5-point Likert scale

Dichotomous = Yes/No/Don't know; Agreement from 1 "not at all" to 5 "absolutely", ^A ^= Cronbach's Alpha

Data was gathered from 142 subjects from 14 different EU member states. All subjects participated in the World Conference on Tobacco or Health in Helsinki in August 2003. With this, we assumed that respondents were at least somewhat knowledgeable in the field of tobacco control. At the place of registration to the conference participants were randomly contacted by research assistants and were handed out the questionnaire and its internet-address. They were asked to fill out the 40-item questionnaire as paper pencil on the site of the conference or could fill out a version online afterwards. The online version was made available to reach more respondents. Additionally, all participants who provided their e-mail address in the conference participants book were addressed via e-mail afterwards. Among the participants, 52% were female, 19% indicated that their field of work was in education, 28% in treatment, 30% in research and 23% in policy. Confidence in their answers was "very confident" for 36.6% of the respondents, 55.2% were "quite confident", 6.2% "not very confident", 0.7% "not confident at all" and 1.4% did not indicate their confidence. Subjects with missing data and subjects "not confident at all" were excluded from the analysis.

The statistical analysis was restricted to calculating raw scores of each subscale per country and the average rank of a country across all subscales and all countries. Items belonging to one subscale were summed and then divided by the number of items of this subscale. The prevention subscale was calculated differently, because the experts felt that just indicating whether there is a legal regulation does not describe prevention strategies appropriately. Therefore the questions about compliance were added and the subscale was calculated as follows: If participants indicated that there was a regulation (question 4 or 5), this response was calculated with "1", when compliance was reinforced with a "5" on the Likert scale (resp. 0.8 when it was 4, 0.6 when it was 3, 0.4 when it was 2 and 0.2 when it was 1). The range of item 6 was transformed accordingly. From all respondents of one country an average raw score could be calculated for each subscale. With this information countries were ranked in each subscale (not shown in the table) and the average rank of a respective country across all subscales was calculated (shown in Table 2). This procedure is more appropriate than summing up the raw scores of all items, since items of different subscales might correlate negatively.

**Table 2:** Results of the MAToC on a population of 142 subjects from 14 different EU-member states

country	n	Mean rank	Prevalence Adult smoking **	Tax Range: 0–1	Smuggling Range: 0–1	Prevention Range: 0–1	Product Control Range: 0–1	Smoking Cessation Range: 1–5	ETS Range: 1–5	Media Support Range: 1–5	Health care system Range: 1–5	Research Range: 1–5	Politics Range: 1–5	Population Range: 1–5
Finland	28	2.4	23%	.83	.60	.83	.87	3.58	4.40	3.31	3.98	3.00	2.94	3.58
Sweden	14	3.6	17.4% *	.43	.43	.69	.88	3.45	3.79	3.57	4.39	2.83	2.79	3.86
Ireland	6	4.3	31%	.83	.67	.72	.67	3.29	3.67	3.00	4.25	2.81	2.99	3.25
UK	24	4.8	28% *	.79	.38	.75	.85	3.78	2.68	3.13	3.57	2.87	2.62	2.89
Netherlands	14	6.3	33.2%	.36	.14	.63	.81	3.76	3.27	2.98	3.21	2.82	2.61	3.18
Italy	4	7.4	31.1% *	.25	.50	.69	.92	1.88	3.00	2.83	3.00	1.92	2.83	2.88
France	6	7.5	27%	.75	.67	.18	.78	3.50	3.33	2.94	2.83	2.11	2.35	2.67
Denmark	12	7.6	30%	.33	.42	.40	.78	3.54	2.94	2.83	3.17	2.36	1.92	2.96
Austria	6	8.7	29%	.33	.17	.78	.89	3.04	2.93	2.06	3.00	1.28	1.79	2.33
Spain	13	9.0	39.1% *	.25	.43	.65	.85	2.61	2.37	2.33	3.07	2.43	2.13	2.11
Belgium	6	9.4	28% *	.33	.17	.36	.83	2.79	3.03	2.33	3.08	1.89	1.36	2.17
Portugal	1	9.5	29.4% *	1	0	1	.67	1.25	2.00	2.00	3.00	1.67	2.33	2.50
Greece	2	10.7	46.8% *	.25	0	.21	1	2.63	2.60	1.83	3.00	1.83	1.50	1.50
Germany	6	11.5	34.5%	.33	0	.72	.83	2.33	1.90	1.72	2.00	1.47	1.38	1.83

Footnote: the countries are sorted by their mean rank across the different dimensions of tobacco control activities; the range in columns 5 to 7 indicates the reference points of the dimensions: 0–1 with 0 = No and 1 = Yes; the range in columns 8 to 15 indicates the reference points 1–5 with 1 = "not all", 5 = "absolutely"; * = these figures represent male adult smoking, siince smoking rates for the general population were not available; ** = percentage smokers in the adult population, taken from the WHO-report for the European Region [1]; ETS= Protection from Exposure to Environmental Tobacco Smoke, due to the differences in participants per country no variance measures were calculated

## Results

Subjects from the tobacco field in Finland gave the highest TC values to their country indicating that Finland was the most active in TC among the countries in our sample, followed by Sweden, Ireland, the UK and the Netherlands (Table 2). The least active countries in TC were Greece and Germany, behind Austria, Spain, Belgium and Portugal. Italy, France and Denmark constituted the middle field. Table 2 also gives country profiles across the different fields of TC. For example: the UK was ranked 4^th ^overall. While they had a leading position in the field of smoking cessation (an average agreement to the statements about support for smoking cessation of 3.78, with 1 indicating no agreement at all and 5 indicating absolute agreement), they put less effort into the protection from ETS (an average agreement of 2.68) when compared on the European level. In this fashion each country shows its own, individual profile, and countries at the end of the ranking also have dimensions where they are European average on TC or even better. For example, Germany was rated last in the EU overall, but looking at the dimension of prevention participants from Germany evaluated their country with 0.72, which is comparably high among the EU-member states. By giving average raw scores Table 2 also indicates the size of the difference between certain EU-member states in a certain TC field. For example: Protection from ETS is rated very high in Finland (4.4) and very low in Germany (1.7), while the difference between Ireland and the UK is very small (3.67 and 3.68). Comparisons between the dimensions reveal that the evaluation of activities in research can be improved in all EU-member states (highest score of 3.00) while support by the health care system is estimated fairly high in all EU-member states (lowest score 2.00). Table 2 also indicates prevalences for smoking in the adult population. Due to the small number of participants and to the differences in quality of the smoking rates more sophisticated analyses of the relation between smoking prevalence and different TC activities were not possible [5].

## Discussion

The MAToC can be answered easily and quickly, so that its application fields are large samples of respondents. Further research with it seems an economic way to assess TC in European countries.

The questionnaire developed may provide a profile of TC across European countries by indicating benchmarks for countries such as illustrated here. It also indicates the actual amount of TC in a specific area in a specific country as it is perceived by experts from the tobacco field. This information is valuable for each country in terms of where they stand with their efforts in TC in comparison to other countries as well as where there is still room for improvement in their own country. What does this mean for specific countries? Germany, for example, is ranked lowest in TC overall. Looking at specific dimensions one can see that in Germany protection from ETS is perceived the worst in Europe by far. German efforts in ETS could benefit from looking into Finnish efforts in this field, since Finland is the leading country in this dimension. In prevention on the other hand, Germany seems to be on an average European level. Another example is Finland: they can benefit from the information provided by MAToC concerning research efforts. Even though Finland is leading in comparison to the other states, the raw score in this dimension leaves room for improvement. By assessing data with this instrument longitudinally changes in TC can be evaluated. Institutions like the European Monitoring Centre for Drugs and Drug Addiction (EMCDDA) in Lisbon could use such an instrument after it has been psychometrically tested.

The results are plausible since they fit into the findings which exist so far. The findings correspond to the amount of TC provisions. Furthermore, they correspond to those of Fagerstrom et al. [6] according to where Finland and Sweden found to be among the highest ranks and Germany and Austria found to be among the lowest ranks with regards to the anti smoking climate. Also, looking at the smoking prevalence taken from the WHO-report.^1 ^the 2 countries with the highest prevalence (Spain, Greece) are ranked very low, the ones with the lowest prevalence are ranked the highest in TC (Finland, Sweden). But since the mean rank is an aggregated value the relation between prevalence and specific TC dimensions needs to be analysed in more detail, for example with the development of multi-dimensional models. This could not be achieved by this analysis because of the small number of respondents. Further validation and examination of psychometric properties of the MAToC is necessary.

The data presented here fulfils the purpose to illustrate what is possible with such an instrument. Yet, there are some limitations to this study: (a) The psychometric properties of the instrument need to be examined. While the face-validity is high, other forms of validity have yet to be tested empirically. We assume that the participants we chose have a valid picture of TC in their country, but further research should validate the MAToC by comparing it with other instruments and by examining the relationship between smoking prevalence and TC as was previously done for the US [7]. Additionally a validation could also include trend in lung cancer development, number of ex-smokers and sales of cessation products. However, this could not be achieved by this study since it is the first study to quantify a wide scope of TC, and to our knowledge no other comparable instrument exists. Furthermore to the lack of comparable instruments there also is a lack of comparable data about smoking prevalence due to different definitions. Re-test reliability needs to be examined so that this instrument can be used to assess longitudinal changes and inter-rater-reliability needs to be examined to give a picture how well the instrument measures different perceptions of experts in each country. These analyses will also provide information, e.g. whether a subscale consisting of just one item (like smuggling) is valid and reliable. (b) Even though we tried to raise the number of people participating, the number of experts per country still differs and is partly rather small. This might be due to a lack of TC experts in some countries. Since representativity was not the main goal of this study this question needs to be addressed in further research with this tool. Future research needs to identify organisations that provide a good amount of experts, so that a larger scale study can be carried out with the MAToC. (c) The items regarding regulation of advertisement needed to be excluded. However this topic represents an important field of tobacco control and should be included in the revised version of the MATOC. Then it should be similar to the prevention items, asking whether there is a regulation towards restriction of advertising followed by an item where the participants can indicate how comprehensive this is. (d) Even though we assume that the experts chosen were knowledgeable about TC in their country this instrument does not measure the actual level, but the respondent's perception and knowledge about TC. To measure the actual level of TC different instruments need to be developed. Results might differ and further research could compare perceptions and actual levels and their relation better.

We conclude that the approach used for this study is valuable in delivering the information wanted. This brief, easy to fill out questionnaire can be used to compare TC activities across the EU countries like they are evaluated by experts from the tobacco field. Benchmarking of EU-member states regarding TC in general and in specific areas is possible and can deliver clear cut information to support political decision making. This procedure could serve as model of practice for other areas like alcohol or legal drug control in the EU, too. Assessing different areas of control policy could lead to the comprehensive description of drug control, working patterns of policies could be identified and policy making could be tailored to country-specific needs. However, the present analyses is just a first step in the area of tobacco control.

## Keypoints

• This scale provides a quantitative ranking of European countries indicating their perceived activity in various fields of tobacco control and relates them to smoking prevalence.

• Decision makers and advocates get an overview on differences across country to help and support them in developing future plans.

• The tool is a first step in quantifying tobacco control, further research is needed to optimize and improve measurement of tobacco control.

## Competing interests

The author(s) declare that they have no competing interests.

## Authors' contributions

Dr. Jochen René Thyrian conducted the project, the survey, and the analyses and took the leading part in drafting the manuscript.

Prof. Ulrich John contributed significantly to the theoretical background and the discussion of the results.

Both authors jointly developed the study design and approved the final draft of the manuscript.
